# *Passiflora cincinnata* Extract Delays the Development of Motor Signs and Prevents Dopaminergic Loss in a Mice Model of Parkinson's Disease

**DOI:** 10.1155/2017/8429290

**Published:** 2017-08-01

**Authors:** Luiz Eduardo Mateus Brandão, Diana Aline Morais Ferreira Nôga, Aline Lima Dierschnabel, Clarissa Loureiro das Chagas Campêlo, Ywlliane da Silva Rodrigues Meurer, Ramón Hypolito Lima, Rovena Clara Galvão Januário Engelberth, Jeferson Souza Cavalcante, Clésio Andrade Lima, Murilo Marchioro, Charles dos Santos Estevam, José Ronaldo Santos, Regina Helena Silva, Alessandra Mussi Ribeiro

**Affiliations:** ^1^Universidade Federal do Rio Grande do Norte, Natal, RN, Brazil; ^2^Universidade Federal de Sergipe, São Cristóvão, SE, Brazil; ^3^Universidade Federal de São Paulo, São Paulo, SP, Brazil; ^4^Universidade Federal de São Paulo, Santos, SP, Brazil

## Abstract

*Passiflora cincinnata *Masters is a Brazilian native species of passionflower. This genus is known in the American continent folk medicine for its diuretic and analgesic properties. Nevertheless, few studies investigated possible biological effects of* P. cincinnata* extracts. Further, evidence of antioxidant actions encourages the investigation of possible neuroprotective effects in animal models of neurodegenerative diseases. This study investigates the effect of the* P. cincinnata* ethanolic extract (PAS) on mice submitted to a progressive model of Parkinson's disease (PD) induced by reserpine. Male (6-month-old) mice received reserpine (0.1 mg/kg, s.c.), every other day, for 40 days, with or without a concomitant treatment with daily injections of PAS (25 mg/kg, i.p.). Catalepsy, open field, oral movements, and plus-maze discriminative avoidance evaluations were performed across treatment, and immunohistochemistry for tyrosine hydroxylase was conducted at the end. The results showed that PAS treatment delayed the onset of motor impairments and prevented the occurrence of increased catalepsy behavior in the premotor phase. However, PAS administration did not modify reserpine-induced cognitive impairments. Moreover, PAS prevented the decrease in tyrosine hydroxylase immunostaining in the* substantia nigra pars compacta* (SNpc) induced by reserpine. Taken together, our results suggested that PAS exerted a neuroprotective effect in a progressive model of PD.

## 1. Introduction

The genus* Passiflora* (Passifloraceae) comprises species noted by their edible fruits, exotic flowers, and use in folk medicine for sedative, anxiolytic, diuretic, and analgesic effects [1–5]. The phytochemical profile of the species of this genus is complex. Phenols, cyanogenic glycosides, alkaloids, and flavonoids can be found in their composition and can be responsible for their pharmacological effects [2]. These compounds could also be related to biological activities of these plants such as anti-inflammatory [6], sedative [7], antihyperglycemic [8], antiulcer [9], anxiolytic [10–13], and antioxidant [9, 14–16] actions. David and colleagues [17] reported higher antioxidant action and lower toxicity of the* Passiflora cincinnata* methanolic extract when compared to other plants from Brazilian* Caatinga*. In addition, Wondracek and colleagues [18] detected carotenoids compounds such as neoxanthin, trans-violaxanthin, antheraxanthin, lutein, zeaxanthin, and trans-*β*-carotene in the* P. cincinnata* fruit pulp. In this respect, carotenoids can modulate intracellular signaling cascades associated with inflammatory cytokines and antioxidant enzymes production. These modulatory activities lead to antioxidant, antiapoptotic, and anti-inflammatory effects that may represent an important improvement in the treatment of neurodegenerative disorders [19].

Neurodegenerative disorders such as Parkinson's and Alzheimer's diseases show severe social and economic challenges, with high impact on public health systems around the world. PD is a progressive and degenerative neurological pathology, characterized by neuronal loss in multiple brain regions, but mostly dopaminergic neurons in the* substantia nigra pars compacta* (SNpc) [20, 21]. Additionally, the neuropathology of this disease is characterized by the formation of intraneuronal protein clusters of *α*-synuclein, referred to as Lewy's bodies [22–24]. The degeneration in SNpc cells and consequent dopaminergic depletion in the striatum result in the classic motor symptoms of PD: resting tremor, rigidity, postural instability, and bradykinesia [20, 25, 26]. This depletion may be result of an imbalance between the production of prooxidants (e.g., reactive oxygen species) and endogenous antioxidant agents (e.g., catalase and glutathione), which generates cellular machinery damage, leading to events such as endoplasmic reticulum or mitochondrial dysfunctions, protein degradation, and apoptosis [27].

Dopaminergic medications used in the treatment of patients with Parkinson's disease are associated with motor and nonmotor behavioral side effects. The dopamine precursor 3,4-dihydroxyphenylalanine (levodopa or L-dopa) is the most efficient treatment to control motor deficits of PD patients [28, 29]. However, patients treated with levodopa (up to 80%) develop side effects such as dyskinesia and motor fluctuations due to the on-off effect [29–31]. In addition, current available treatments do not reduce or preclude neurodegeneration [32, 33].

Over the years, the use of animal models to evaluate neurochemical and neuropathological aspects of PD has been critical to the understanding of PD's etiology, as well as the validation of potential treatments. The chronic administration of a low dose of reserpine (0.1 mg/kg, s.c.) has been proposed as a progressive pharmacological model of PD in rats [34] and mice [35], mainly because this protocol gradually provokes motor and nonmotor impairments mimicking the progressive nature of the PD symptoms [36]. The alkaloid reserpine induces monoamine depletion, oxidative stress, inflammation, proapoptotic commitment, reduction in tyrosine hydroxylase levels, increase in *α*-synuclein immunostaining, and upregulation of DA receptors [36]. In addition, this protocol induces progressive motor impairments preceded by cognitive deficits [35, 37], which is consistent with the general development of the disease in humans. The aim of the present study was to evaluate the effects of the ethanolic extract of* Passiflora cincinnata* on motor, cognitive, and neuronal parameters in mice submitted to repeated treatment with reserpine.

## 2. Material and Methods

### 2.1. Ethanolic Extract Preparation

Leaves of* Passiflora cincinnata* were collected in Moita Bonita city, Sergipe state, Brazil. Professionals from the University of Sergipe Herbarium identified the specimen. A voucher specimen (ASE 11,112) has been deposited in the herbarium of the institution for reference. After identification, samples were dried at 37°C in an oven with air renewal and airflow for 48 h until complete dehydration. The material was crushed with a knife mill and subsequently powdered (293.3 g). Afterwards, it was extracted by maceration at room temperature in 90% ethanol for 5 days. The extract was filtered in vacuum, and the solvent was removed using a rotary evaporator (45°C) under reduced pressure and freeze-dried, yielding the ethanolic extract of the PAS (EEPc). The percentage of EEPc yield was 35.2%.

### 2.2. Animals

Six-month-old male Swiss mice were housed in groups of 6–8 per cage (30 × 37 × 16 cm) under conditions of acoustic isolation, controlled airflow and temperature (25 ± 1°C), and a 12 h light/dark cycle (lights on 6:30 a.m.) with food and water available ad libitum. Animals used in this study were handled in accordance with Brazilian law for the use of animals in research (Law Number 11.794) and the local ethics committee for animal usage approved all the procedures (Protocol CEUA/UFRN number 003/2013). All efforts were made to minimize animal pain, suffering, or discomfort during treatment.

### 2.3. Drugs Treatment and General Procedures

Reserpine (RES, Sigma Chemical Co., USA) was dissolved in glacial acetic acid (1%) and then diluted to the correct concentration with distilled water. Vehicle consisted of the same amount of acetic acid and water as in the reserpine solution. Both reserpine and vehicle were injected subcutaneously (s.c.) in a volume of 10 mL/kg.

Before the beginning of experimental procedures, animals were gently handled for 10 min for 5 consecutive days. The apparatuses were cleaned with 5% alcohol solution after each behavioral session and all behavioral data were registered and analyzed by the video-tracking software Any-maze (Stoelting, USA), except for the catalepsy test and the oral movement's evaluation that were manually registered by researchers blind to treatment.

### 2.4. Experimental Design

Mice were randomly assigned to one of four groups: CTR/CTR (*n* = 13), CTR/PAS (*n* = 15), RES/CTR (*n* = 17), and RES/PAS (*n* = 16). Animals received subcutaneous injections of vehicle (CTR) or 0.1 mg/kg of reserpine (RES) at a volume of 10 mL/kg body weight, every 48 h for 40 days of treatment. Moreover, mice received daily intraperitoneal injections of PAS vehicle (CTR) or 25 mg/kg of extract at a volume of 10 mL/kg body weight for 40 days. Animals did not show signs of intoxication, weight lost, pain, or discomfort during the treatment, showing good biological tolerability of the PAS ethanolic extract.

**Figure 1:**
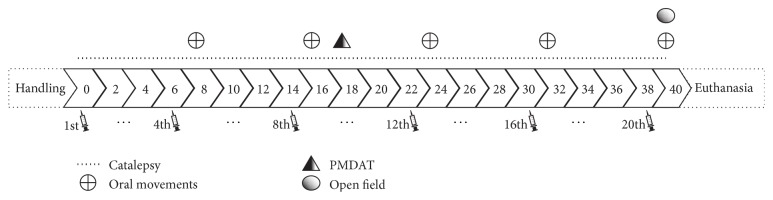
Schematic representation of the neuroprotective evaluation of PAS administration in mice.

Animals were submitted to the following procedures before the daily injections (between 8:00 a.m. and 4:00 p.m.): (1) catalepsy test; (2) assessment of oral movements 48 h after the 4th, 8th, 12th, 16th, and 20th injections; (3) discriminative avoidance task 24 h before (training) and after (testing) the 10th injection; (4) evaluation of open field behavior 48 h after the 20th injection. Experimental design is shown in Figure 1.

### 2.5. Behavioral Tests

#### 2.5.1. Catalepsy Test

The catalepsy behavior was assessed by placing the animal's forepaws on a horizontal bar positioned 5 cm above the bench surface. Catalepsy was defined as an immobile posture (keeping both forepaws on the bar) and was measured up to a maximum of 180 s. Three trials per animal in each observation day were carried out, and the results were analyzed considering the mean value of these trials.

#### 2.5.2. Oral Movements

Mice were individually placed in a transparent glass box (20 × 20 × 15 cm) with mirrors positioned under and behind it to allow behavioral quantification when the animal faced away from the observer. The frequency of vacuous chewing movement (mouth openings in the vertical plane not directed toward physical material) and duration (s) of twitching of the facial musculature were measured continuously for 10 min.

#### 2.5.3. Plus-Maze Discriminative Avoidance Task (PMDAT)

The apparatus employed is a modified elevated plus-maze, made of wood, containing two open arms (27.5 × 6.5 cm) opposite to two enclosed arms (27.5 × 6.5 × 18 cm). One lamp and one loudspeaker were placed over one of the enclosed arms (aversive enclosed arm). In each side of the apparatus, there were different extramaze visual cues that animals could use to distinguish the location of different arms of the maze. Two behavioral sessions were performed in each experiment. During the training session, mice were placed individually in the center of the apparatus facing the open arms intercept and, over 10 min, every time animals entered in the aversive enclosed arm they received aversive stimuli until leaving the arm. These aversive stimuli were noise (80 dB) and light (100 W). The test session was carried out 24 h after the training session. In this test, mice were again placed in the apparatus for 5 min, without receiving any aversive stimulation. Animals were considered to be in a certain arm when the four paws passed over its entrance [38]. In both sessions, the amount of time spent in each enclosed arm (aversive and nonaversive) was registered and compared in each group for learning and memory evaluation. Moreover, locomotor activity and anxiety-like behavior were evaluated by total distance travelled in the apparatus and percentage of time spent on open arms (% TOA, time spent in open arms divided by the summation of time spent in open and enclosed arms), respectively. Further, in the training session, ethological components of risk assessment were evaluated by the following parameters: head dipping (frequency of movements of the head toward the floor), categorized into protected head dipping (PHD, when performed from the center of apparatus) and unprotected head dipping (UHD, when performed from the open arms), and stretched attend postures (SAP, defined by stretching and contraction of body to its original position without locomotion).

#### 2.5.4. Open Field

At the final day of the protocol animals were submitted to the open field in order to evaluate locomotor and exploratory activities. The apparatus was a circular arena (50 cm in diameter) with 40 cm high walls, made of wood and painted black. Animals were placed in the center of the apparatus for free exploration during 10 min. Distance travelled in the whole arena (m), average speed (m/s), and time spent in the center of the open field (s) were evaluated.

#### 2.5.5. Tissue Processing

Upon completion of the behavioral procedures, animals were deeply anesthetized with intraperitoneal injection of thiopental sodium (100 mg/kg) and perfused transcardially with 100–150 mL phosphate-buffered saline (PBS), pH 7.4, containing 0.2% heparin, followed by 150 mL PBS with 4% paraformaldehyde 0.1 M. The brains were removed from the skull and postfixed in the same fixative solution previously described and stored at 4°C. After 24 hours, we transferred the brains to a solution containing sucrose 30% 0.1 M PBS, at 4°C. Each brain was fixed in Tissue-Tek® (Sakura, Japan) at −20°C. Then we serially sliced the brains in the coronal plane into 30 *μ*m thick sections with a cryostat microtome (Leica, Germany) at a temperature of −20°C.

#### 2.5.6. Tyrosine Hydroxylase (TH) Immunohistochemistry

Following tissue processing, we performed immunohistochemistry for TH, using a free-floating protocol. Sections were washed out 4 times with PBS (pH 7.4) for 5 min each and consecutively washed with 0.3% H_2_O_2_ solution for 20 min to reduce endogenous peroxidase activity. For the detection of TH, sections were incubated with rabbit anti-tyrosine hydroxylase polyclonal antibody (cat # AB152 Chemicon, USA, 1 : 10,000). The antibody was diluted in triton x-100 0.4% and PBS with 2% albumin serum, for 18–24 h at room temperature. Afterwards, sections were incubated with goat biotinylated anti-rabbit IgG (Vector Labs, USA, 1 : 5,000) diluted with triton x-100, 0.4% NaCl, and PBS for 2 h at room temperature. Then, a new washout process was carried out followed by an incubation with avidin-biotin-peroxidase solution (ABC Elite kit, Vector Labs, Burlingame, USA). The reaction was developed by adding of 3,3-diaminobenzidine (DAB, Sigma-Aldrich, USA) and 0.01% H_2_O_2_ 0.1 M phosphate buffer solution for 1-2 min. Then, we left sections to dry, dehydrated in a graded alcohol series, cleared in xylene, and coverslipped with Entellan (Merck). All sections were immunostained concomitantly, to minimize possible background differences between samples. Sections were examined under brightfield illumination with an optical microscope (Nikon Eclipse Ni-E), attached with a digital camera (Motic 5.0) to record images.

In order to estimate the number of TH+ cells in SNpc and TH levels in striatum, four sections of each animal were selected for each region evaluated (SNpc and striatum, *n* = 4–7 per group): one at the rostral level, two at medium level, and one at caudal level, representative of the rostrocaudal extension of each area of interest. The exact location of the regions was determined on the basis of the Paxinos and Franklin [39] mice brain atlas. These sections were chosen by a systematic sampling and all measurements were performed in a blind manner. All TH+ cells of SNpc on each section were counted and the mean of the four measures was registered. Additionally, TH+ levels in striatum fibers were assessed by analysis of relative optical densitometry (ROD), using ImageJ software (version 1.48, NIH, USA). For this purpose, we transform our images in 8-bit color grade (i.e., grayscale), and four random fields were chosen in the target area (dorsal striatum). The mean values of gray level in the target areas were subtracted from the mean value of a control region (used to assess “noise” or nonspecific staining, i.e., cortex or* corpus calosum*). Finally, all values were normalized considering the control group mean value, in order to evaluate proportional alterations.

### 2.6. Statistical Analysis

Data normality and the homogeneity of variances were, respectively, tested by the Shapiro-Wilk and Levene's tests. All comparisons among groups for locomotor parameters and anxiety-like behaviors from PMDAT were performed by one-way ANOVA followed by Dunnett's test, whereas learning and memory parameters were analyzed by the paired-samples *t*-test. Catalepsy behavior and oral movements were compared between groups across treatment period using ANOVA with repeated measures followed by Tukey's test. In the open field test, parameters were compared between groups using one-way ANOVA followed by Tukey's test. Results were expressed as mean ± SEM and *p* < 0.05 was considered to reflect significant differences.

## 3. Results

### 3.1. Catalepsy Behavior

**Figure 2:**
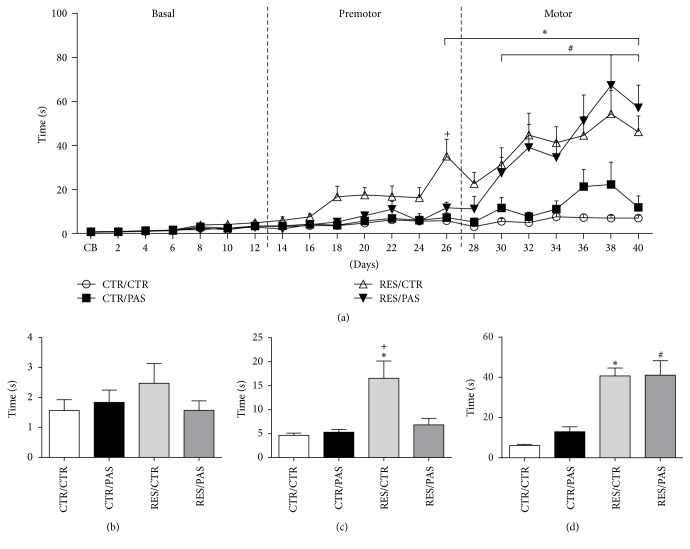
Effects of repeated administration of* Passiflora cincinnata* extract (25 mg/kg) and reserpine (0.1 mg/kg) on catalepsy behavior of mice. (a) Entire treatment analysis, (b) basal phase, (c) premotor phase, and (d) motor phases. Data are expressed as mean ± SEM. ^*∗*^*p* < 0.05 RES/CTR compared to CTR/CTR; ^#^*p* < 0.05 RES/PAS compared to CTR/CTR; and ^+^*p* < 0.05 RES/CTR compared to RES/PAS (repeated measures ANOVA followed by Tukey's post hoc test).

ANOVA with repeated measures revealed time versus treatment interaction [*F*_(20,60)_ = 6.365, *p* < 0.001]. Post hoc analysis showed that repeated treatment with reserpine induced progressive increase in the duration of catalepsy behavior, with RES-treated animals being significantly different from control group from the 26th (RES/CTR) and the 30th (RES/PAS) days onwards (see Figure 2(a)). No differences were found considering chronic administration of PAS per se (CTR/PAS). To clarify the differences between the groups we subdivided the treatment length in three phases: basal (beginning of procedures to 12th day), premotor (14th to 26th day), and motor (28th to 40th day) phases. This new analysis, now subdivided by phases, revealed time × treatment interactions for premotor [*F*_(6,18)_ = 4,942, *p* < 0.001] and motor phases [*F*_(6,18)_ = 3,534, *p* < 0.001]. Post hoc analysis revealed significant increased catalepsy in RES/CTR compared to CTR/CTR and RES/PAS groups in the premotor phase (Figure 2(c)). In the motor phase both RES/CTR and RES/PAS groups showed increased catalepsy time when compared to CTR/CTR group (Figure 2(d)).

### 3.2. Oral Movements

**Figure 3:**
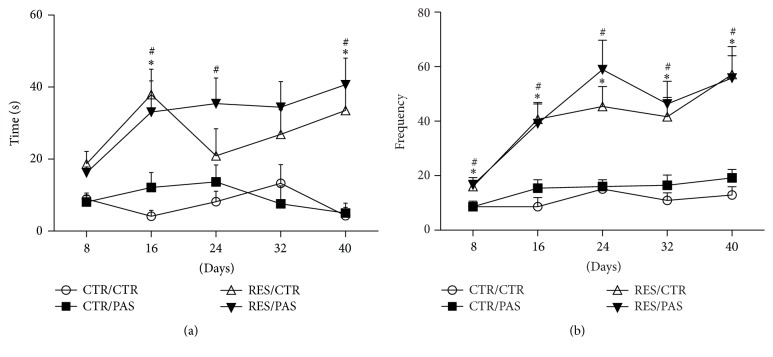
Effects of repeated administration of* Passiflora cincinnata* extract (25 mg/kg) and reserpine (0.1 mg/kg) on oral movements of mice. (a) Twitching and (b) vacuous chewing. Data are expressed as mean ± SEM. ^*∗*^*p* < 0.05 RES/CTR compared to CTR/CTR; ^#^*p* < 0.05 RES/PAS compared to CTR/CTR (repeated measures ANOVA followed by Tukey's post hoc test).

ANOVA with repeated measures revealed effect of treatment [*F*_(3,55)_ = 14.112, *p* < 0.001] for duration of oral twitching. We found a significant increase in RES/CTR group when compared to CTR on the 16th (48 h after the 8th injection, *p* < 0,01) and 40th (48 h after the 20th injection, *p* < 0,01) days, and RES/PAS group showed increase in 16th, 24th, and 40th days (*p* < 0,01) (Figure 3(a)).

For number of vacuous chewing movements, ANOVA with repeated measures revealed time × treatment interaction [*F*_(4,12)_ = 2.247, *p* < 0.05]. Indeed, animals receiving reserpine (RES/CTR and RES/PAS) showed a significant increase when compared to control groups in all assessment days (Figure 3(b)).

### 3.3. Plus-Maze Discriminative Avoidance Task (PMDAT)

No differences were found in total distance travelled in the training [*F*_(3,26)_ = 1.708, *p* = 0.193] and test [*F*_(3,26)_ = 1.672, *p* = 0.201] sessions (Table 1).

No differences were found in % TOA [*F*_(3,26)_ = 0.407, *p* = 0.749], UHD [*F*_(3,26)_ = 1.291, *p* = 0.301], and PHD [*F*_(3,26)_ = 2.171, *p* = 0.119]. However, one-way ANOVA revealed an increase in CTR/PAS values of SAP [*F*_(3,26)_ = 3.175, *p* < 0.05] when compared to the CTR/CTR group (Table 1).

**Figure 4:**
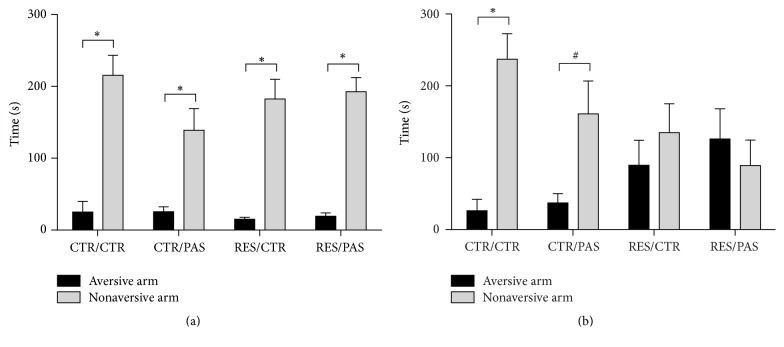
Effects of repeated administration of* Passiflora cincinnata* extract (25 mg/kg) and reserpine (0.1 mg/kg) on mice exploration of the aversive and nonaversive arms in plus-maze discriminative avoidance task. (a) Training session and (b) test session. Data are expressed as mean ± SEM. ^*∗*^*p* < 0.05 and ^#^*p* = 0.07 compared to aversive arm (paired-samples *t*-test).

In the training session, paired-samples *t*-test showed that all groups spent more time in the nonaversive enclosed arm indicating that all animals learned the task [CTR/CTR: *t*_(3)_ = 4.501, *p* < 0.05, CTR/PAS: *t*_(5)_ = 3.090, *p* < 0.05, RES/CTR: *t*_(7)_ = 6.404, *p* < 0.001, and RES/PAS: *t*_(8)_ = 7.903, *p* < 0.001] (Figure 4(a)). However, in the test session, only CTR/CTR and CTR/PAS groups remembered the learned task [CTR/CTR *t*_(3)_ = 4.107, *p* < 0.05, CTR/PAS *t*_(5)_ = 2.302, *p* = 0.07] (Figure 4(b)).

### 3.4. Open Field Test

**Figure 5:**
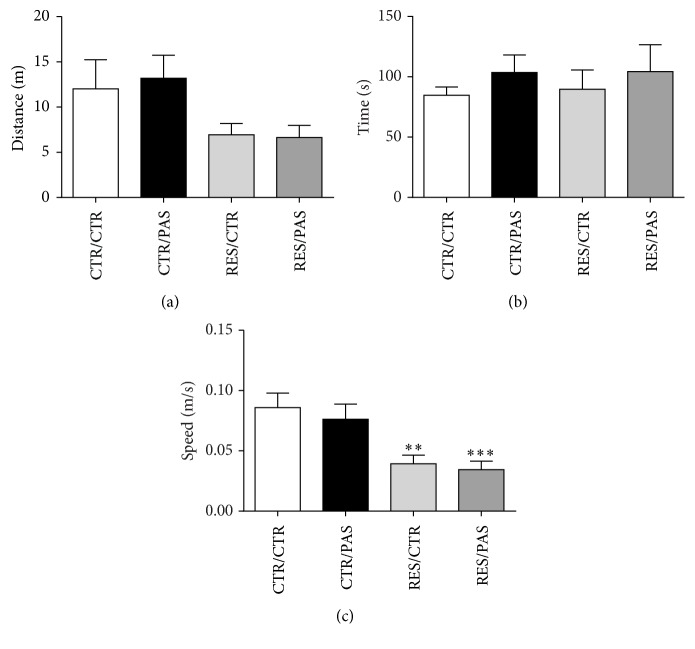
Effects of repeated administration of* Passiflora cincinnata* extract (25 mg/kg) and reserpine (0.1 mg/kg) on (a) total distance travelled, (b) time spent in central zone, and (c) average speed of mice in open field. Data are expressed as mean ± SEM. ^*∗∗*^*p* < 0.01 and ^*∗∗∗*^*p* < 0.005 compared to control (one-way ANOVA followed by Tukey's post hoc test).

In this test, no differences were found for total distance travelled [*F*_(3,55)_ = 2.670, *p* = 0.057] and time spent in the center of open field [*F*_(3,55)_ = 0.322, *p* = 0.809]. However, reserpine groups (RES/CTR and RES/PAS) showed a decrease in the average speed [*F*_(3,55)_ = 7.152, *p* < 0.001] (Figure 5).

### 3.5. Tyrosine Hydroxylase Immunohistochemistry

**Figure 6:**
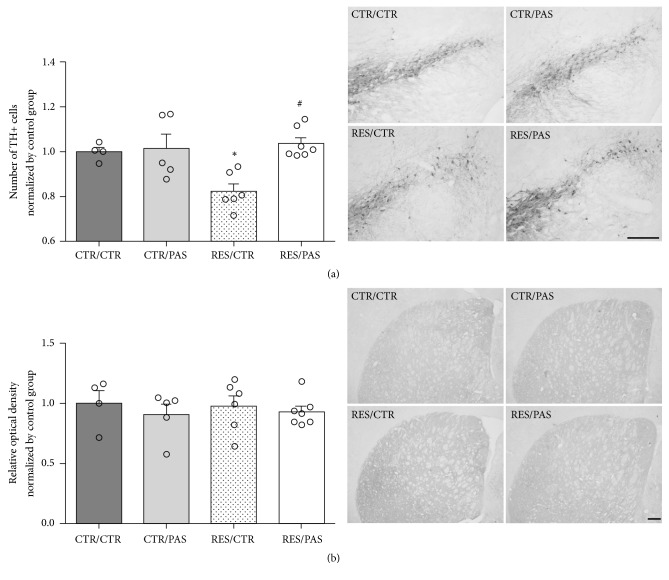
Effects of repeated administration of* Passiflora cincinnata* extract (25 mg/kg) and reserpine (0.1 mg/kg) on (a) TH+ cells of SNpc and (b) relative optical density (ROD) of dorsal striatum, both normalized by CTR values. Data are expressed as mean ± SEM. ^*∗*^*p* < 0.05 compared to CTR/CTR. ^#^*p* < 0.05 compared to RES/CTR. (one-way ANOVA followed Tukey's post hoc test). Magnification 100x (a) and 40x (b), black bold lines are scale bars, corresponding to 200 *μ*m.

For the number of TH+ cells in SNpc, one-way ANOVA revealed significant differences between groups [*F*_(3,21)_ = 7.329, *p* < 0.005]. Post hoc analysis revealed a decrease in the number of TH+ cells on RES/CTR when compared to CTR/CTR group as well as an increase on RES/PAS when compared to RES/CTR group (Figure 6(a)). No differences were found in relative optical density of dorsal striatum [*F*_(3,21)_ = 0.268, *p* = 0.847] (Figure 6(b)).

## 4. Discussion

In this study, we investigated the effects of the administration of the ethanolic extract of* P. cincinnata* on reserpine-induced parkinsonism. Our main results showed that mice chronically treated with PAS displayed a delayed onset of motor impairments induced by reserpine, but the treatment did not modify reserpine-induced cognitive impairment. In addition, concomitant PAS treatment prevented the depletion of TH+ SNpc cells caused by the chronic administration of reserpine.

Reserpine administration induces depletion of monoamines by blocking vesicular monoamines transporters (VMATs), which results in motor disturbances like tremor, rigidity, and hypokinesia [40–42]. This blockage of VMATs generates a cytoplasmic accumulation and further decrease of neurotransmitters release. Moreover, monoamines left in cytoplasm are metabolized, generating reactive metabolites, which leads to oxidative stress [43–46]. Thus, it seems to be an appropriate animal model for development of new drugs for treatment of PD [47, 48].

The majority of studies using reserpine as a PD model focus on a single high dose administration [49–52]. In an attempt to mimic the PD's progressive profile, a recent study from our group demonstrated that chronic administration of a low dose of reserpine was able to induce progressive motor impairment, accompanied by lipid peroxidation due to oxidative stress [34] and tyrosine hydroxylase depletion in dorsal striatum and SNpc [37]. In the present study, we used an adaptation of this protocol to mice, as described by Campêlo and colleagues [35]. Specifically, there is an increase in the treatment duration; that is, the number of injections was altered from 10 to 20 (during 40 days). This adaptation is necessary because mice are more resilient to reserpine than rats. A possible explanation to this resilience is the fact that mice have less monoamine oxidase (MAO) activity in the brain compared to rats [53], which could contribute to a lower formation of oxidative metabolites from dopamine degradation (e.g., hydrogen peroxide). Furthermore, these physiologic differences may also be responsible for the minor decrease in TH+ SNpc cells (this reduction is more expressive in rats), and consequently no reduction of striatal densitometry, which might have mitigate possible PAS effects (Figure 6(b)). We suggest that this result may be related to a compensatory mechanism. In this respect, previous studies have reported that remaining dopaminergic neurons in SNpc could sustain a more intense expression of TH to replace the new dopamine demand in their terminals, converting more tyrosine into dopamine [54]. This new dopamine demand would occur in response to reserpine action on VMAT, leading to the sustained expression of TH densitometry levels reported in dorsal striatum of reserpine groups.

Interestingly, the administration of the ethanolic extract of* P. cincinnata* delayed the onset of the motor impairment (increased catalepsy behavior) induced by reserpine treatment. Indeed, while RES/CTR group showed motor deficits from the 26th day after the beginning of treatment, the impairment was present in the RES/PAS group only from the 30th day onwards (Figure 2). Furthermore, the coadministration with PAS also prevented the tyrosine hydroxylase depletion in the SNpc cells, which occurred in the RES/CTR group (Figure 6(a)). Nevertheless, reserpine-treated animals also showed reduction in average speed in open field locomotion (Figure 5(c)) and increased oral twitching and vacuous chewing movements (Figure 3), which were not prevented by PAS cotreatment.

Some studies have demonstrated the neuroprotective activity of antioxidant compounds in animals treated with reserpine. The antioxidant substances (e.g., ebselen and vitamins E and C) reduced oxidative stress parameters such as thiobarbituric acid reactive substances (TBARS) and catalase levels [55–57]. In this context, an earlier research showed that* P. cincinnata* has an antioxidant activity [17]. Based on this report, we speculate another mechanism underlying this antioxidant effect. It is known that flavonoids may trigger an internal cellular response through the activation of the PKC/ARE/Nrf2 pathway [58]. This signaling promotes transcription of NAD(P)H:quinone oxidoreductase-1 (NQO1) and other detoxifying genes [59], which is impaired by reserpine treatment because it decreases PKC activity [60]. Consequently, we could infer that this characteristic is responsible for the delay in the onset of motor deficits in the catalepsy test, as well as the decrease in tyrosine hydroxylase depletion in SNpc cells.

As mentioned above, in this PD model a more reduction in TH+ SNpc cells is observed in rats than in mice. Therefore, the PAS effects in the striatum would be better detectable if mice showed an expressive reduction of TH+ cells. In other words, it is possible that different metabolism rates between species may have overshadowed the results regarding TH levels in the striatal dopaminergic projections. Nevertheless, it the TH+ cell count in the SNpc did reduce after reserpine treatment, and PAS was able to prevent it.

Regarding the oral movements evaluation, it is important to highlight the differences found in the sensitivity of both parameters to the effects of reserpine treatment. Both parameters were able to detect motor deficits in the beginning of the treatment, even before the appearance of catalepsy increment. However, the effect of time and the interaction between time and treatment were only observed for vacuous chewing, a motor alteration well established in the literature as a consequence of reserpine treatment [34, 55, 61–63]. Regardless, development of those impairments in our animals were not prevented or delayed by PAS treatment.

Regarding cognitive evaluation in the PMDAT, we did not observe any changes in learning, since all groups were able to discriminate the aversive and the nonaversive arms during the training session (Figure 4(a)). On the other hand, in the test session, reserpine groups had a retrieval deficit, which was not affected by cotreatment with PAS (Figure 4(b)). The memory impairment in animals that received reserpine corroborates previous data from our group [64, 65] and other reports [66, 67]. This impairment might be compared to recognition or evaluation deficits present in patients with PD. Studies proposed that these changes are linked to an imbalance in basal ganglia dopamine availability. This imbalance affects circuit connections to regions related to cognitive and emotional functions, like prefrontal cortex, amygdala, hippocampus, and ventral tegmental area, among other regions [68–71]. Importantly, similar to the evaluation of oral movements, cotreatment with PAS did not alter reserpine-induced memory deficit. Taken together, these two findings suggest that the protocol of PAS treatment used here was not fully effective in preventing all alterations related to reserpine-induced parkinsonism.

In addition, although PAS treatment did not improve other parameters, the positive effects observed in catalepsy behavior and tyrosine hydroxylase expression are relevant because they suggest a potential delay in the neurodegeneration caused by PD. The use of this chronic treatment with a low dose of reserpine, a well established model for PD [36], positively contributed to demonstrating the subtle effect of PAS that might not be evidenced in acute treatment with neurotoxins (the usual method of PD inducement in rodent models). We believe that a preventive (i.e., before reserpine injections) treatment with PAS, increased doses of the extract, or longer treatments could have a more widespread effect. Alternatively, the application of fractions as well as isolated* P. cincinnata* compounds could also be more effective.

Regarding possible effects on anxiety-like behavior, no differences were found in relation to percentage of time in open arms (Table 1), but studies have demonstrated that the evaluation of risk assessment behavior (stretched attend posture and head dipping) may be useful in anxiety evaluation [72, 73]. In the present study, we observed an increase in the anxiety level (stretched attend postures frequency, Table 1) in animals treated with PAS extract (training session), and this effect could be responsible for the slight performance reduction presented in memory retrieval by this group, although it was not significantly different from control (Figure 4(b)).

## 5. Conclusions

In summary, this research suggests that the ethanolic extract of* P. cincinnata* has neuroprotective properties that may have therapeutic potential for PD. Based on the literature, this effect is probably related to an antioxidant-related action (see above). However, further studies are required to assess the range of these effects regarding parkinsonian symptoms, as well as to determine the structure of active compounds and their mechanisms of action.

## Figures and Tables

**Table 1:** Effects of repeated administration of *Passiflora cincinnata* (25 mg/kg) and reserpine (0.1 mg/kg) on total distance travelled (training and test sessions) and anxiety-like parameters (training session) in plus-maze discriminative avoidance task.

Treatment	Total distance travelled (meters)		Anxiety-like parameters (frequency)			
	Training	Test	% TOA	SAP	PHD	UHD
CTR/CTR	11.82 ± 2.99	9.41 ± 4,04	7.27 ± 2.74	26.25 ± 2.50	5.75 ± 1.38	8.00 ± 3.70
CTR/PAS	14.23 ± 2.50	9.56 ± 3.24	19.58 ± 5.63	42.50 ± 4.06^*∗*^	14.17 ± 4.44	24.00 ± 9.47
RES/CTR	9.64 ± 1.20	4.96 ± 1.68	18.92 ± 10.80	38.38 ± 3.02	6.63 ± 1.66	10.38 ± 3.22
RES/PAS	9.41 ± 1.09	4.18 ± 0.86	14.13 ± 4.52	35.44 ± 2.76	8.22 ± 1.28	17.78 ± 5.27

Data expressed as mean ± SEM. ^*∗*^*p* < 0.05 compared to CTR/CTR (one-way ANOVA followed by Tukey's post hoc test). % TOA: percentage of time spent on open arms, SAP: stretched attend postures, PHD: protected head dipping, UHD: unprotect head dipping.
